# Eco-friendly spark-generated Co_x_O_y_ nanoparticle-modified graphite screen-printed sensing surfaces for the determination of H_2_O_2_ in energy drinks

**DOI:** 10.1007/s00604-024-06233-3

**Published:** 2024-02-22

**Authors:** Maria Siampani, Alexandros Ch. Lazanas, Konstantinos Spyrou, Mamas I. Prodromidis

**Affiliations:** 1 Department of Chemistry, University of Ioannina, 451 10 Ioannina, Greece; 2 Department of Materials Science & Engineering, University of Ioannina, 451 10 Ioannina, Greece

**Keywords:** Spark generated nanoparticles, Green method, Cobalt nanoparticles, Hydrogen peroxide electrode, Amperometry, Food analysis

## Abstract

The modification of graphite screen-printed electrodes (SPEs) is reported using an eco-friendly and extremely fast method based on the direct cobalt pin electrode-to-SPE spark discharge at ambient conditions. This approach does not utilize any liquids or chemical templates, does not produce any waste, and allows the *in-situ* generation of Co_x_O_y_ nanoparticles onto the electrode surface and the development of efficient electrocatalytic sensing surfaces for the determination of H_2_O_2_. Co-spark SPEs were characterized using scanning electron microscopy, energy-dispersive X-ray spectroscopy and x-ray photoelectron spectroscopy (XPS), revealing the formation of surface confined Co_x_O_y_ nanoparticles and the diverse oxidation states of cobalt species. Co-spark SPEs were also characterized with cyclic voltammetry and electrochemical impedance spectroscopy. Redox transitions of the surface confined electrocatalysts are demonstrated by electrochemical polarization studies, showing the formation of different oxides (Co_x_O_y_), varying the XPS results. Amperometric measurements at 0.3 V vs. Ag/AgCl revealed a linear relationship between the current response and the concentration of H_2_O_2_ over the range 1 − 102 μM, achieving a limit of detection (3σ/m) of 0.6 μM. The interference effect of various electroactive species was effectively addressed by employing dual measurements in the absence and presence of the enzyme catalase. The analytical utility of the method was evaluated in antioxidant rich real-world samples, such as energy drinks, demonstrating sufficient recovery.

**Graphical Abstract:**

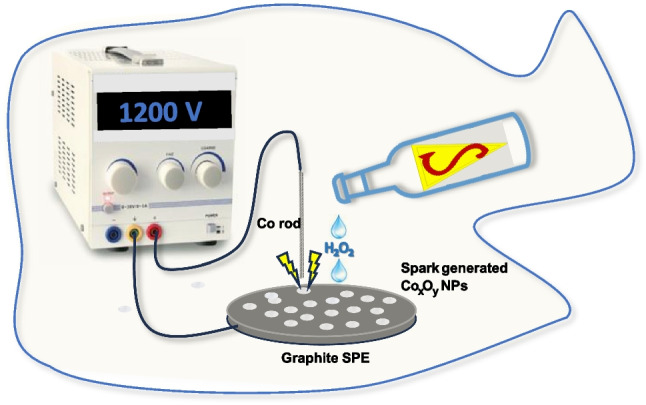

**Supplementary Information:**

The online version contains supplementary material available at 10.1007/s00604-024-06233-3.

## Introduction

Energy drinks is a group of carbonated beverages that has grown significantly popular over the past two decades. Their basic ingredients are caffeine (up to 0.04% w/v), carbon dioxide and other components such as sugars, salts, taurine, amino acids and B-complex water-soluble vitamins [1]. They also contain various reducing species to suppress the oxidation process caused by oxygen, oxygen derived species (ROS) and free radicals [2]. While the adverse effects of ROS and free radicals are usually regulated by multiple protective responses in vivo, the chemistry of mixtures of redox-active ingredients and oxygen is relatively unregulated in formulated food products, particularly aqueous beverages, such as energy drinks [2, 3]. In the presence of oxygen and any oxidisable compounds acting as substrates, hydrogen peroxide (H_2_O_2_) can be generated by progressive reduction of molecular oxygen, and consequently, there is the possibility of its ingestion, in the case of beverages and foods. Over the past years there have been reports of the production of H_2_O_2_ in various foods and drinks. The production of H_2_O_2_ has been verified in beer brewing, originating from L-cysteine and thiol-rich proteins respectively [4]. Another instance is the production of H_2_O_2_ in polyphenolic beverages like cocoa [5], green [6–9] and black tea [7, 9], red wine [8], and similar phenolic-rich drinks under physiological conditions.

The non-enzymatic electrochemical determination of H_2_O_2_ can be achieved through its oxidation at (noble) metal electrodes at high overpotentials (> + 0.65 V versus common reference electrodes) [10]. However, this method may lead to a loss of selectivity in the presence of other reducing species and fouling phenomena, gradually reducing the response of the electrodes [10]. Alternatively, H_2_O_2_ can be reduced under sufficiently cathodic potentials, typically in deoxygenated solutions to mitigate interference from the oxygen reduction reaction [9].

To address these limitations, a significant amount of research has been dedicated to the modification of electrode surfaces with various charge-transfer mediators [11–13], electrocatalysts [14–16], conducting polymers [17, 18], biomolecules [19–21], noble metal (such as platinum, and gold) nanoparticles (NPs) [9, 22–24] etc. The current emphasis is on the advancement of chemical sensors employing non-noble (like copper, nickel, iron, and cobalt) metal NPs, particularly through eco-friendly methods that eliminate the need for organic solvents and costly reagents. This approach aligns with the principles of green chemistry and is geared towards reducing sensor costs [25–30].

Among the non-precious metal based electrocatalysts for H_2_O_2_, spinel type (A^2+^B_2_^3+^X_4_^2−^, where A^2+^and B_2_^3+^ are metal cations and X_4_^2−^ are usually chalcogens such as oxygen [31–34] or sulfur [35, 36]), nano structures of ferromagnetic elements (Fe, Co and Ni), which present redox pairs that can effectively mediate the electro oxidation or reduction of H_2_O_2_, have been also proposed [31, 33–36].

Our study details the *in-situ* modification of graphite screen-printed electrodes (SPEs) using cobalt oxide nanoparticles (Co_x_O_y_ NPs) through an eco-friendly spark-discharge process. This process occurs between a cobalt pin electrode and the graphite SPE, leading to instant surface modification and advanced electrocatalytic properties towards H_2_O_2_. The direct modification of the electrode surface with spark-generated nanoparticles represents a robust approach in line with environmental sustainability considerations and the principles of green chemistry in modern electroanalysis [30]. This method has been acknowledged as highly effective for developing various sensing surfaces (such as Mo NPs [37], Au NPs [38, 39], Ag NPs [40], carbon NPs and nanosheets [41–43]) with a simple, liquid-free, and cost-effective procedure. The effective modification of graphite SPEs with Co_x_O_y_ NPs is substantiated through scanning electron microscopy (SEM), x-ray photoelectron spectroscopy (XPS), cyclic voltammetry, and electrochemical impedance spectroscopy (EIS). Furthermore, the analytical efficacy of Co-spark SPEs for the determination of H_2_O_2_ in energy drinks is demonstrated.

## Experimental

### Materials

A cobalt piece (Sigma-Aldrich, 99.5% trace metal basis) was fine cut with a metal saw to narrow strips to be used as cathode material (electrode pin) in the sparking process. Before use, the strips were thoroughly rinsed and sonicated with acetone. Sodium hydroxide, sodium dihydrogen phosphate, potassium chloride, ascorbic acid, caffeine and D-glucose were purchased from Merck. Hexaammineruthenium(III) chloride (RuHex) was purchased from Aldrich. Catalase from bovine liver (EC 1.11.1.6, ≥ 200 KU mL^−1^) was a Fluka product. A stock solution of ca. 0.1 M H_2_O_2_ was prepared by appropriate dilution of the stock product (30% H_2_O_2_, Supelco) in double distilled water (DDW), stored at 4 °C, and was weekly standardized with the permanganate method. Working solutions were daily prepared by appropriate dilutions of the stock solution in DDW.

### Apparatus

Electrochemical measurements were conducted with an Autolab PGSTAT12/FRAII electrochemical analyser (Metrohm Autolab) in a conventional 3 − electrode cell. Plain or cobalt sparked SPEs (Co-spark SPEs) were used as the working electrode, while a Ag/AgCl 3 M KCl electrode (IJ Cambria) and a platinum wire served as the reference and the counter electrode, respectively. All the potential values quoted are referred to the potential of the reference electrode.

Cyclic voltammograms (CVs) were recorded in 0.1, 0.5 or 1 M NaOH at a scan rate of 0.05 V s^−1^ (unless stated otherwise). EIS spectra were recorded in 0.5 M NaOH over the frequency range from 100 kHz to 0.1 Hz using a sinusoidal excitation signal of 10 mV (rms) amplitude superimposed on a DC potential of 0.120 V or 0.500 V. Amperometry measurements were conducted in stirred (300 r.p.m) solutions of 0.5 M NaOH at 0.3 V. XPS measurements were conducted under ultrahigh vacuum with a base pressure of 2 × 10^−9^ mbar using a SPECS GmbH instrument equipped with a monochromatic MgKa source (hv = 1253.6 eV) and a Phoibos-100 hemispherical analyzer. The energy resolution was set to 1.18 eV and the photoelectron take-off angle was 45° with respect to the surface normal. Recorded spectra were set with energy step set of 0.05 eV and dwell time of 1 s. All binding energies were referenced with regard to the C1s core level centered at 284.6 eV. Spectral analysis included a Shirley background subtraction and peak deconvolution involved mixed Gaussian–Lorentzian functions was conducted with a least squares curve-fitting program (WinSpec, University of Namur, Belgium). Field-emission scanning electron microscopy (FE-SEM) images were taken with a Phenom Pharos G2 desktop FEG-SEM (ThermoFisher Scientific) at 11 kV on chromium coated samples (Quorum Q150T ES plus, sputter coater).

### Fabrication and modification of electrode surface

The *in-situ* modification of the graphite SPE surface with spark-generated Co_x_O_y_ NPs was implemented using a 16 × "linear" sparking mode. This involved connecting the cobalt electrode pin as the cathode ( −) and the graphite SPE as the anode ( +) to a high-voltage power supply. The two electrodes were brought into proximity (approximately 1 mm) through a G-code-controlled 2D positioning device until spark discharge occurred at 1.2 kV DC under ambient conditions. An external capacitor (2.8 nF) was connected in parallel to the power supply output terminals. Details on the experimental setup for electrode modification with electrical discharge and the fabrication of the graphite SPE can be found in Refs. [38, 41, 42] and Ref. [40], respectively. The electroactive area (*A*) of the plain and Co-spark SPE was calculated using double potential step chronocoulometry in 1 mM RuHex in 0.1 M KCl according to the procedure given in Ref. [44].

### Analytical procedure

Energy drink samples were purchased at the local market. The samples were degassed in an ultrasonication bath for 10 min, and then were used to prepare the following solutions: (A) 1.0 mL sample, 0.5 mL 1 M phosphate buffered saline (PBS) pH 6, and 0.5 mL 2 M NaOH; (B) 1.0 mL spiked sample (950 μL sample plus 50 μL 50 mM H_2_O_2_), 0.5 mL 1 M PBS pH 6, and 0.5 mL 2 M NaOH; (C) 1.0 mL sample, 0.5 mL enzyme solution (480 μL 1 M PBS pH 6 plus 20 μL catalase), and, after the hand mixing of the solution for 10 min, 0.5 mL 2 M NaOH. In sample (C), PBS pH 6, is used to maximize the enzymatic activity of catalase [45], while in samples (A) and (B), which do not contain catalase, PBS was added to ensure that the same assay protocol was applied for all the measurements. Catalase was employed to eliminate H_2_O_2_, and as a result, the signal of solution (C) is ascribed to the electroactive species coexisting in the sample. Consequently, it was subtracted from the signals of solutions (A) and (B).

Amperometric measurements were conducted under stirring in an electrochemical cell containing a 2.0 mL aliquot as described for solutions A, B, or C, and 8.0 mL 0.5 M NaOH. The concentration of H_2_O_2_ in both the plain and spiked samples was determined by applying the standard addition method.

## Results and discussion

### Morphological studies

**Fig.1:**
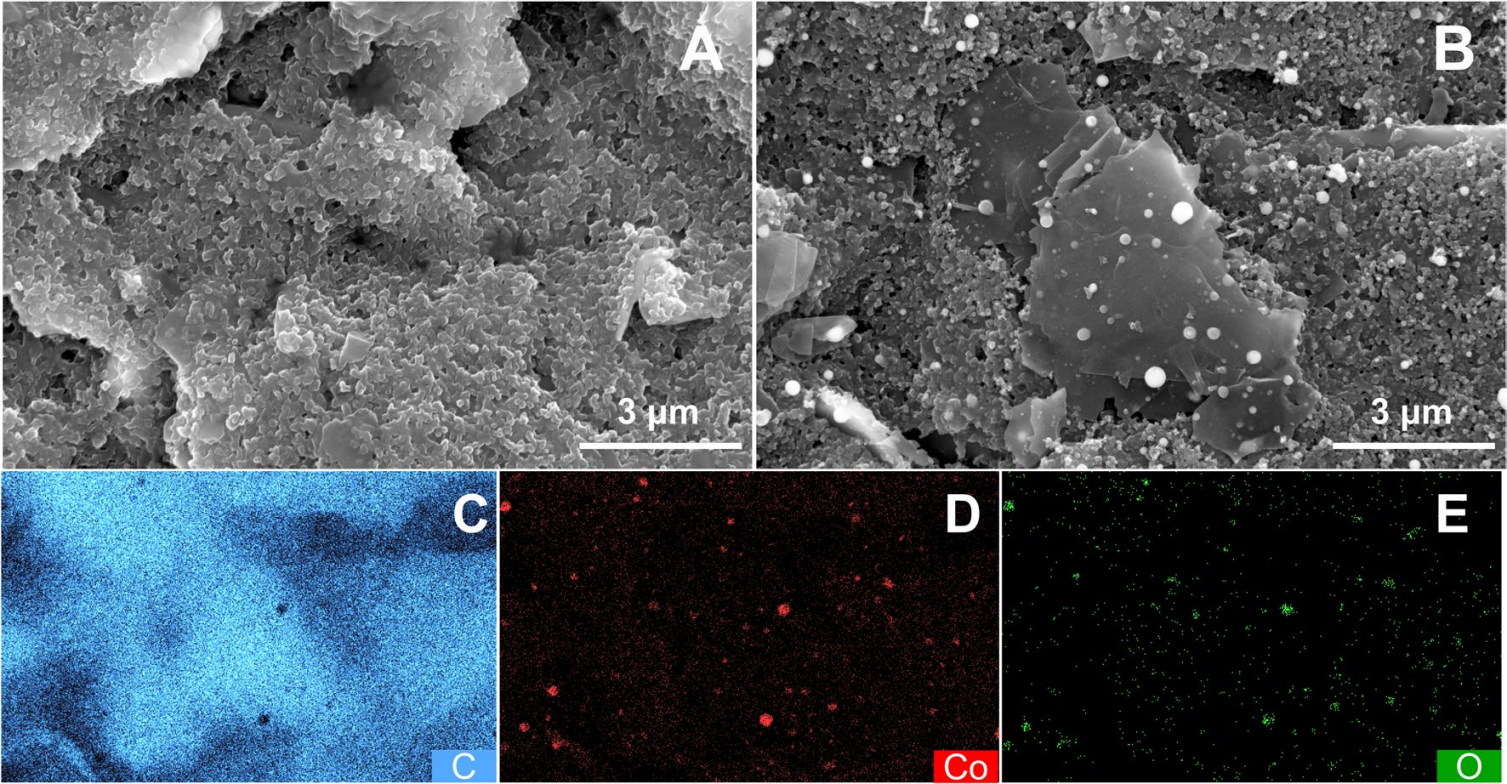
SEM images of (**A**) plain and (**B**) Co-spark SPE. EDS mapping of (**C**) carbon, (**D**) cobalt and (**E**) oxygen atoms on the surface of Co-spark SPE

SEM images of plain and Co-spark SPE are shown in Fig. 1A and B, respectively. It is apparent that while the plain SPE has a compact layered structure of graphite, the Co-spark SPE shows exfoliated, micrometre-sized graphite sheets enriched with spherical cobalt nanoparticles. This double impact of the spark process benefits the sparked electrode in terms of sensitivity due to the electrocatalytic effect steaming from the cobalt-based NPs and the augmentation of the electroactive surface area of the SPE due to exfoliated nanosheets produced. Based on chronocoulometric measurements in 1 mM RuHex in 0.1 M KCl, the electroactive area of plain and Co-sparked SPEs [44], was found to be 0.1126 cm^2^ and 0.1883 cm^2^, respectively. From the EDS mapping shown in Figs. 1C-E, the spark generated nanoparticles exhibit both cobalt (depicted with red colour in Fig. 1D) and oxygen (depicted with green colour in Fig. 1E) sites which demonstrate the formation of Co_x_O_y_ NPs on the electrode surface. The average particle diameter of Co_x_O_y_ NPs was found to be 163 ± 73 nm.

### XPS studies

**Fig. 2:**
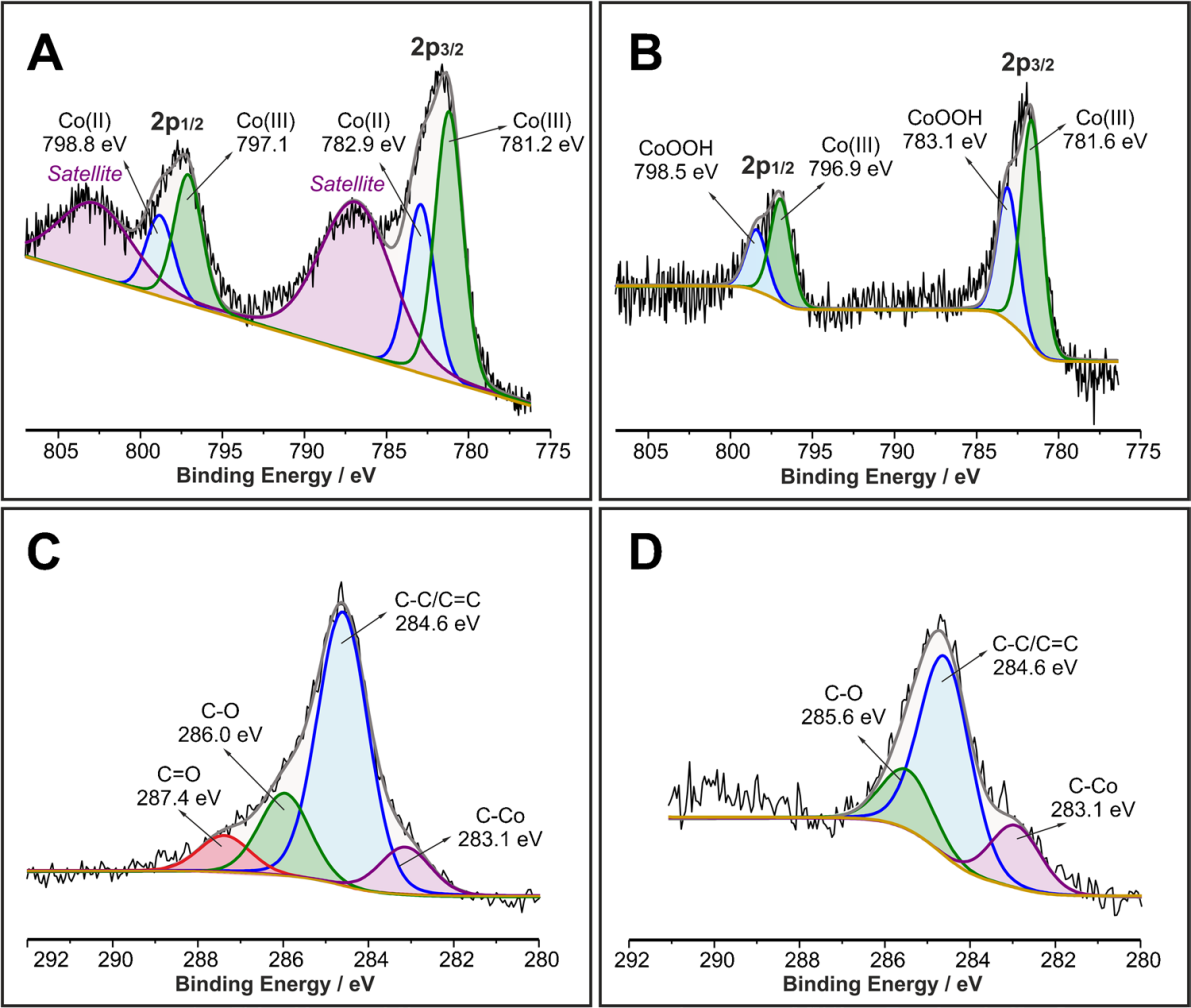
The Co $2p$ spectrum of (**A**) Co-spark SPE and (**B**) Co-spark SPE after five cyclic voltammetry scans from 0 to 0.7 V in 0.5 M NaOH. The C $1s$ spectrum of (**C**) Co-spark SPE and (**D**) Co-spark SPE after five cyclic voltammetry scans from 0 to 0.7 V in 0.5 M NaOH

XPS studies were conducted in the surface of a Co-spark SPE (Figs. 2A, C) and a Co-spark SPE after its treatment with five cyclic voltammetry scans between 0.0 and 0.7 V in 0.5 M NaOH (termed Co-spark SPE-NaOH) (Figs. 2B, D). The Co $2p$ spectrum involves two main peaks corresponding to $2{p}_{1/2}$ and $2{p}_{3/2}$ spin orbitals. The binding energies of $2{p}_{1/2}$ and $2{p}_{3/2}$ are separated by 15.3 eV at Co-spark SPE and 15.8 eV at Co-spark SPE-NaOH, indicating a difference of 0.5 eV for the two electrodes. At the Co-spark SPE, the $2{p}_{3/2}$ orbit is deconvoluted into two peaks at 781.2 eV and 782.9 eV corresponding to the presence of Co(III) and Co(II) species, respectively. The existence of the intense shake-up satellite located at 787.0 eV is attributed to the high-spin nature of Co(II) species [46]. In accordance with previous works [46–48], the observed peaks and positions indicate the formation of Co_3_O_4_ spinel structure.

In the case of Co-spark SPE-NaOH, shown in Fig. 2B, the two fitted peaks of the $2{p}_{3/2}$ orbit are shifted to higher binding energies (781.6 and 783.1 eV). The shift of the binding energies for the Co-spark SPE-NaOH in combination with the difference of the energy separation of about 0.5 eV between the $2{p}_{1/2}$ and $2{p}_{3/2}$ orbitals at the two sparked electrodes as well as the lack of the shake-up features in the spectrum of Co-spark SPE-NaOH lead to the conclusion that the high-spin phase of Co(II) is present only at the Co-spark SPE. Conversely, we can deduce that both the peaks at 781.6 and 783.1 eV are attributed to low-spin Co(III) species [47]. However, it is evident that the Co(III) peak at 781.6 eV in $2{p}_{3/2}$ orbit at Co-spark SPE-NaOH can be attributed to the same Co(III) species existing also in Co-spark SPE, while the peak at 783.1 eV can be attributed to CoOOH, that is, the product of the OH^−^ adsorption and simultaneous oxidation of CoO (782.9 eV in $2{p}_{3/2}$ orbit at Co-spark SPE) according to the chemical equation, $CoO+{OH}^{-}\rightleftharpoons CoOOH+{e}^{-}$.

The C1s photoelectron peak is deconvoluted into four peaks at Co-spark SPE and three peaks at Co-spark SPE-NaOH, respectively as shown in Fig. 2C, D. The basic carbon frame consists of C − C/C = C bonds, while at lower binding energies, at both samples, a small peak which is attributed to the C − Co bond can also be seen. The formation of the C − Co bond can be explained considering the extremely high temperatures, up to 20000 K [49], grown locally due to the sparking process (XPS spots have been selected on the sparked areas).

### Electrochemical characterization

**Fig. 3:**
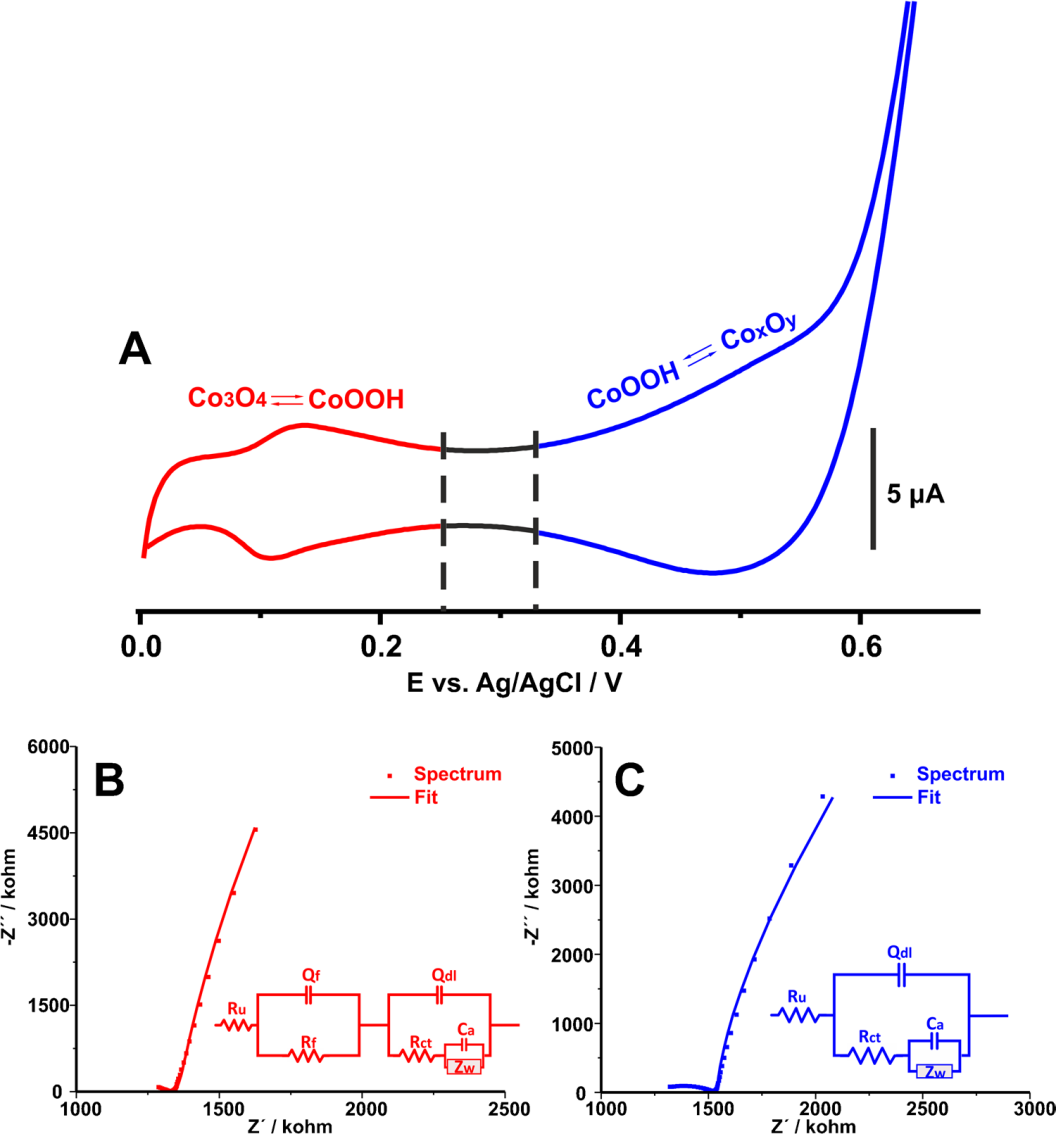
**A** Cyclic voltammogram of Co-spark SPE in 0.5 M NaOH. Scan rate, 50 mV s^−1^. **B** Nyquist plots of Co-spark SPE in 0.5 M NaOH at 0.12 V and **C** 0.5 V. Inset graphs illustrate the respective equivalent electrical circuits depicted at the same coloration

Figure 3A shows the cyclic voltammetric behavior of Co-spark SPE within the potential window from 0 to 0.7 V in 0.5 M NaOH. The recorded CV exhibits two pairs of peaks, which both correspond to quasi-reversible redox transitions. The first redox transition is manifested by a pair of well-defined peaks with a formal potential of ca. 0.12 V, which can be attributed to the following equation:

1 $${{Co}_{3}}^{(II,III)}{O}_{4}+{OH}^{-}+{H}_{2}O\rightleftharpoons 3{Co}^{(III)}OOH+{e}^{-}$$

while the second redox transition is manifested by a pair of broad peaks centered at ca. 0.5 V. According to previous works, this pair of peaks can either be attributed to the complete oxidation of Co(III) to Co(IV) according to Eq. 2 [50, 51]:

2 $${Co}^{(III)}OOH\rightleftharpoons {Co}^{(IV)}{O}_{2}+{H}^{+}+{e}^{-}$$

or to the adsorption of hydroxyl species and the anodic dissolution of the anodic layer [50, 51]. In our case, the fact that no Co^(IV)^O_2_ species were identified in the XPS characterization of the Co-spark electrode after the CV treatment in NaOH, urges us to lean towards the hydroxyl species adsorption explanation.

The effect of scan rate on the cyclic voltammetric behavior of Co-spark SPE was examined with CV measurements at different scan rates from 10 to 500 mV s^−1^ (Fig. S1A). As can be seen in Fig. S1B the peak current values for the first pair of peaks (Ip_a_1, Ip_c_1) have a linear relationship to the square root of the scan rate, indicating a diffusion-limited electrochemical process [52]. Considering that Co_3_^(II,III)^O_4_ molecules are confined onto the electrode, the formation of three molecules Co^(III)^OOH from one Co_3_^(II,III)^O_4_ molecule through a diffusion-limited electrochemical process (Eq. 1) can be explained as follows: Co_3_^(II,III)^O_4_, is a mixed oxide incorporating the Co^(II)^O and Co_2_^(III)^O_3_ forms. Co^(II)^O is oxidized to Co^(III)^OOH through an 1e^−^/OH^−^ mechanism, while at the same time, through the transfer of a H_2_O molecule, one molecule of Co_2_^(III)^O_3_ forms two Co^(III)^OOH molecules [53]. Thus, the CoOOH formation is dependent on the mass transfer (diffusion) of hydroxyl ions and water molecules from the solution to the electrode. Acknowledging the challenge of accurately measuring the current at the second pair of (broad) peaks (Fig. S1A), linear plots between Ip_a_2 and Ip_c_2 with the square root of the scan rate were also received (Fig. S1C), suggesting a diffusion-limited electrochemical process [52]. However, based on the XPS data indicating that Co^(IV)^O_2_ is not formed, the mechanism of this redox transition may be more complex than described by Eq. 2.

In response to the cyclic voltammetric behavior of the Co-spark SPE in alkaline conditions, EIS studies were also conducted by applying either a DC potential of 0.12 V (the formal potential of the Co_3_O_4_/CoOOH redox couple) or 0.5 V (the formal potential of the second redox transition). When the impedance measurements were conducted at 0.12 V and under alkaline conditions, the impedance spectrum (Fig. 3B) exhibited a distorted semicircle over the high frequency range followed by a straight line over the low frequency range. Based on previous studies by Lyons and Brandon [54] regarding the impedimetric behavior of oxide-covered Ni, Co, and Fe electrodes, the obtained impedimetric data were effectively modeled using the equivalent electrical circuit shown as an inset graph in Fig. 3B. The circuit is represented as R_1_(Q_f_R_f_) (Q_dl_[R_ct_(C_a_W)]), where R_1_ represents the electrolyte resistance, (Q_f_R_f_) represent the dielectric properties of the Co_3_O_4_ film [50], Q_dl_ represents the capacitance of the double-layer, R_ct_ represents the charge transfer resistance of the redox transition (Eq. 1), and (C_a_Z_W_) [55] represents the coupled hydroxyl ions diffusion and adsorption, modeling the relaxation of the charge associated with the adsorbed intermediate of the CoOOH phase. As evident from the slope of the linear part of the spectrum over the low-frequency range (slope ≠ 1), the acquired impedance cannot be solely attributed to the semi-infinite diffusion of hydroxyl anions modeled by the Warburg impedance (Z_W_). Instead, it is indicative of a coupled diffusion/adsorption process modeled by Z_W_ and C_a_ components connected in parallel [55].

On the other hand, when the impedance measurements were conducted at 0.5 V and under alkaline conditions, the impedance spectrum illustrated in Fig. 3C can be sufficiently modeled with a quite similar equivalent electrical circuit (Fig. 3C, inset graph). In this case, the (Q_f_R_f_) time constant is not included, which can be interpreted as the complete transition of Co_3_O_4_ to other Co_x_O_y_ species at this potential. Consequently, the final equivalent circuit is R_1_(Q_dl_[R_ct_(C_a_Z_W_)]), where all the symbols have their aforementioned meaning.

### Optimization studies

**Fig. 4:**
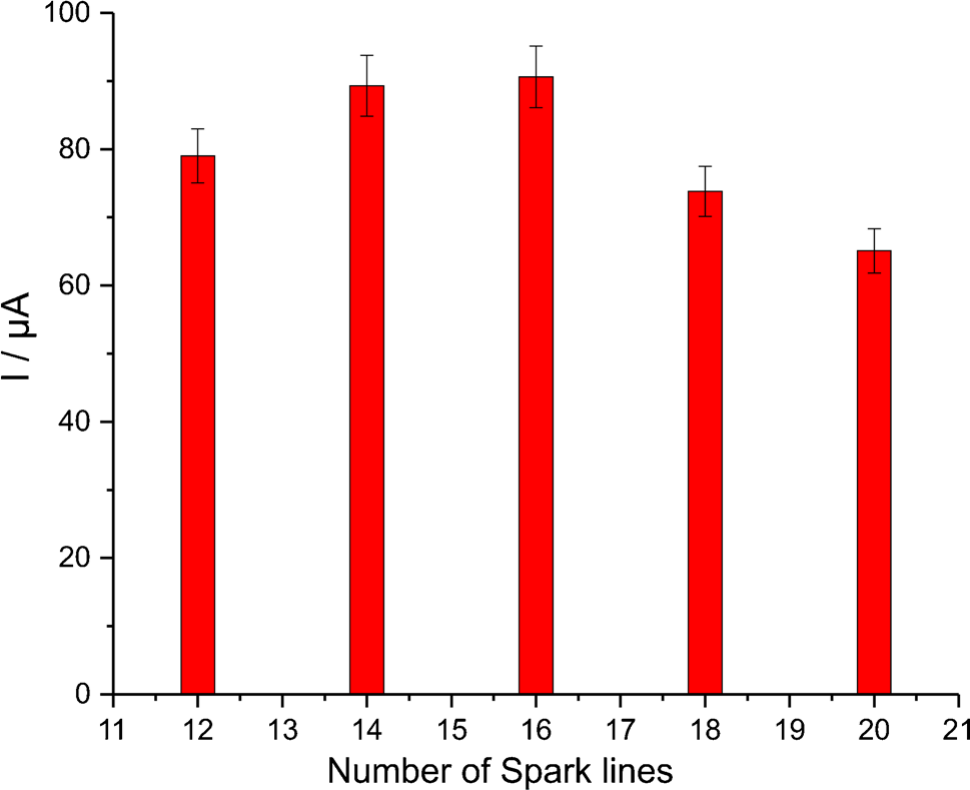
Electrocatalytic currents of Co-spark SPEs modified by a different number of sparking lines (12 − 20) in 0.5 M NaOH, containing 5 mM H_2_O_2_. Errors bars represent the standard deviation of the measurements at three different electrodes

The electrocatalytic activity of the “linear’’ mode Co-spark SPEs towards the electro oxidation of H_2_O_2_ was studied by comparing the cyclic voltammetric responses of SPEs modified with a different number of sparking lines (12, 14, 16, 18, and 20) in the absence and the presence of 5 mM H_2_O_2_. The mean electrocatalytic response and the standard deviation of the measurements with three different electrodes in each case is illustrated in Fig. 4. The highest electrocatalytic responses were observed for a modification of 16 lines, and therefore, Co-spark SPEs modified with 16 sparking lines were selected for subsequent work.

**Fig. 5:**
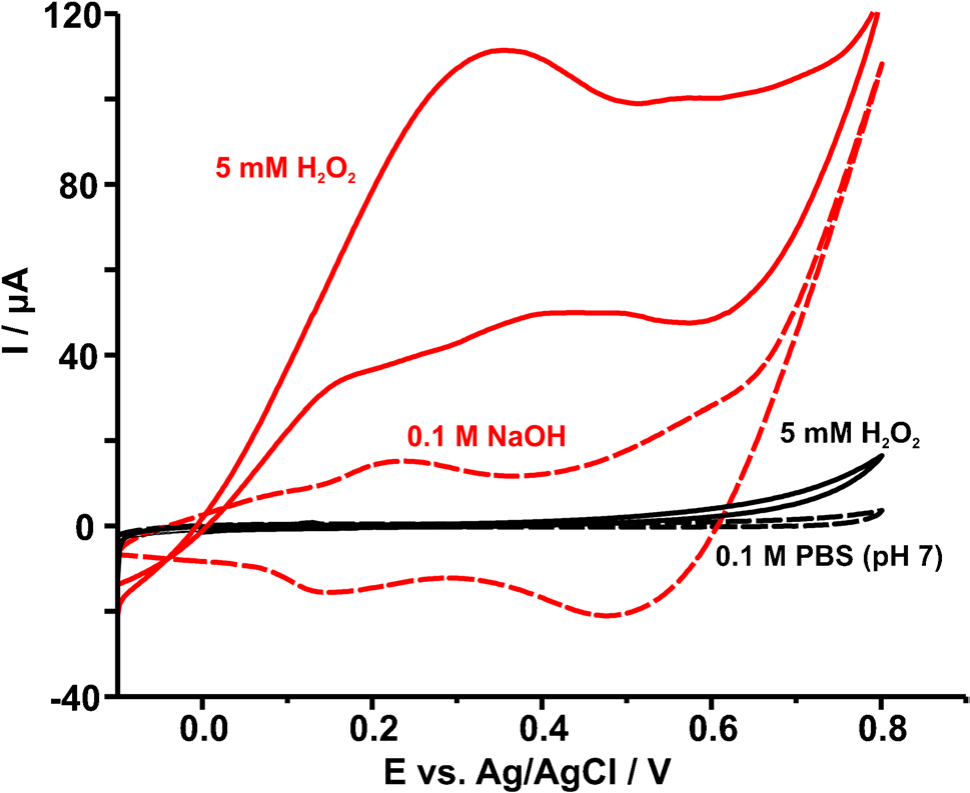
Cyclic voltammograms of Co-spark SPEs in (black line) PBS pH 7 and (red line) 0.1 M NaOH in the (dashed line) absence and (solid line) presence of 5 mM H_2_O_2_. Scan rate 50 mV s^−1^

The effect of the electrolyte on the electrocatalytic activity of Co-spark SPE was also investigated. As shown in Fig. 5, CVs of Co-spark SPE were recorded in 0.1 M phosphate buffered saline (PBS) at pH 7 and 0.1 M NaOH in the absence (dashed line) and presence (solid line) of 5 mM H_2_O_2_. The data revealed a poor electrocatalytic response in neutral pH, while the remarkable electrocatalytic behaviour in alkaline pH seems to be related to the first redox transition Co_3_O_4_/CoOOH and the chemical reduction of the electrochemically generated CoOOH by H_2_O_2_ to Co_3_O_4_, according to Eq. 3. This is then re-oxidized to CoOOH during the sweep (Eq. 1), giving rise to a several-fold increase in the anodic current, while the cathodic current decreases accordingly.

3 $$3{\text{CoOOH}}+{{\text{H}}}_{2}{{\text{O}}}_{2}\to {{\text{Co}}}_{3}{{\text{O}}}_{4}+{{\text{O}}}_{2}+{2{\text{H}}}_{2}{\text{O}}+{{\text{e}}}^{-}+{{\text{H}}}^{+}$$

Consequently, it stands to reason that since the electrocatalytic reaction is mediated by CoOOH species, the concentration of NaOH plays a large role to the electrocatalytic process. The optimum concentration of NaOH was determined by examining the cyclic voltammetry response at 0.1, 0.5, and 1 M NaOH, as illustrated in Fig. S2. In both cases of 0.5 and 1 M NaOH there is a shift of the first redox transition Co_3_O_4_/CoOOH to lower potentials (ca. 0.2 to 0.12 V), compared with that in 0.1 M NaOH, since in those cases there is an abundance of hydroxyl ions facilitating the formation of CoOOH at lower overpotentials. This shift is also prevalent in the presence of 5 mM H_2_O_2_, which favours its electrocatalysis at lower overpotentials as well. However, while both 0.5 and 1 M NaOH enhance the electrocatalytic activity of the modified electrode, the faradaic current produced in the case of 0.5 M NaOH is higher. Therefore, the concentration of 0.5 M NaOH was chosen as the optimum.

### Calibration features

**Fig. 6:**
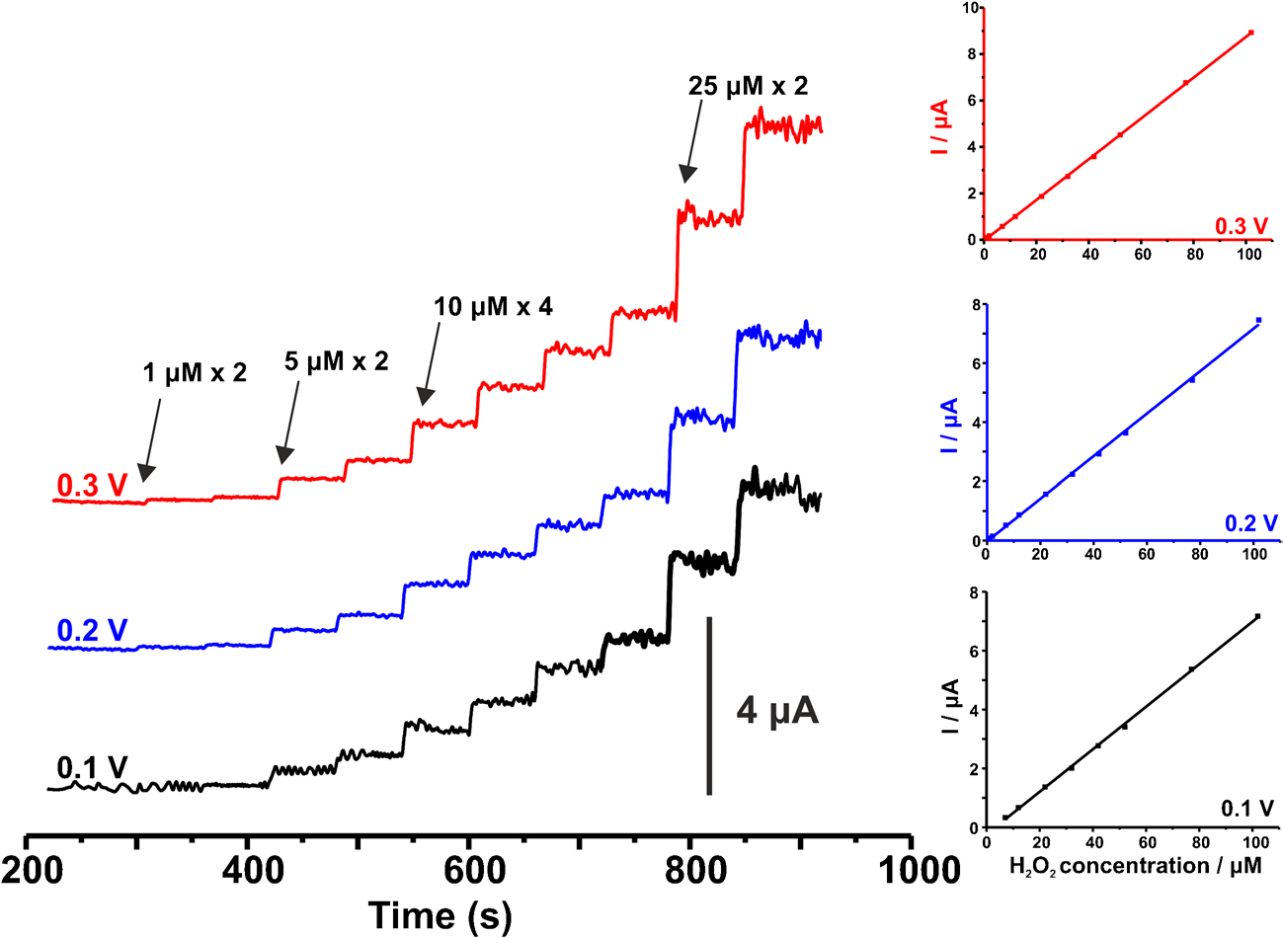
(Left panel) Amperometric plots of Co-spark SPEs over the concentration range 1 – 102 μM Η_2_Ο_2_ at (black line) 0.1, (blue line) 0.2, and (red line) 0.3 V in 0.5 M NaOH. (Right panel) The corresponding calibration plots

The amperometric response of Co-spark SPEs at various concentrations of H_2_O_2_ over the range 1 − 102 μM at three different polarization voltages was investigated. The amperograms at 0.1, 0.2, and 0.3 V and the respective calibration plots are shown in Fig. 6, while the major electroanalytical performance parameters are listed in Table 1. Based on these data and judging by the sensitivity and the limit of detection (LOD), calculated as 3S_a_/slope, at each case, the polarization voltage of 0.3 V was selected as optimum for subsequent work on the determination of H_2_O_2_ in real-world samples.

**Table 1:** Calibration features of Co-spark SPEs for the amperometric determination of H_2_O_2,_ at 0.1, 0.2 and 0.3 V

Voltage (V)	Linear range (μM)	Intercept (10^–8^ A)	S_a_ (10^–8^ A)	Slope (10^–8^ A/μM)	LOD 3S_a_/slope (μM)	R^2^	Sensitivity (μA/μM/cm^2^)
0.1	7 − 102	− 23.5	4.024	7.22	1.7	0.9992	0.383
0.2	1 − 102	− 25.19	3.724	7.19	1.6	0.9989	0.382
0.3	1 − 102	− 4.18	1.841	8.78	0.6	0.9998	0.466

The repeatability of the measurements for three successive additions of 10 μM H_2_O_2_ was found to be 2.9%, while the inter-electrode reproducibility among four different Co-spark SPEs at the concentration level 10 μM H_2_O_2_ was found to be 5.6%. The storage stability of the sensor was also evaluated on a weekly basis by measuring a standard of 10 μM H_2_O_2_, and it was found that Co-spark SPEs maintained more than 85% of their original signal over the course of one month.

Compared with other works on the electrochemical determination of H_2_O_2_ listed in Table 2, Co-spark SPEs exhibit favourable [31, 56–59, 61–63] or comparable [60, 65] analytical features. Considering their low cost, ease of fabrication, and eco-friendliness of modification, it is believed that Co-spark SPEs are highly promising electroanalytical platforms for the determination of H_2_O_2_.

**Table 2:** Comparison of Co-spark SPEs with previously reported H_2_O_2_ sensors

Electrode	Linear range (μM)	Detection limit (μM)	Applicability	Reference
CoFe_2_O_4_ /GO	0.9–900	0.54	Rainwater	[31]
ZnO NSs	1–1000	0.8	H_2_O_2_ released from human hepatoma cells	[56]
Co_3_O_4_ NW/rGO	15–675	2.4	H_2_O_2_ released from liver cancer cells	[57]
CoOOH NSs	4–16	40	−	[58]
CuO-NP/CILE	1–2500	0.5	Milk	[59]
Cu_2_S MC	1–3030	0.2	Serum	[60]
MnO_2_ NW/Gr	100–45000	10	H_2_O_2_ released from live cells macrophage	[61]
Nanoporous PdFe	500–6000	2.1	−	[62]
AuNP-NH_2_/Cu-MOF/GCE	5–850	1.2	H_2_O_2_ released from Hela cells	[63]
WC–Co NP/GCE	0.05–1020	0.0063	Contact lens cleaning solution & human blood	[64]
CuO@Cu_2_O-NW/PVA	1–3000	0.35	−	[65]
Co_x_O_y_ NP/SPE	1–102	0.6	Energy drinks	*This work*

CoFe_2_O_4_/GO, cobalt ferrite/graphene oxide; ZnO NS/zinc oxide nanosheets; Co_3_O_4_ NW/rGO, cobalt(II,III) oxide nanowire/reduced graphene oxide; CoOOH NSs, cobalt oxyhydroxide nanosheets; CuO-NP/CILE, copper(II)oxide nanoparticle/carbon ionic liquid electrodes; Cu_2_S MC, copper(I) sulfide mesoporous carbon; MnO_2_ NW/Gr, manganese(IV) oxide nanowire/graphene; nanoporous PdFe, nanoporous palladium-iron alloy; AuNP-NH_2_/Cu-MOF/GCE, ammoniated gold nanoparticle/copper-based metal oxide framework/glassy carbon electrode; WC–Co NP/GCE, cobalt nanoparticle-decorated tungsten carbide/glassy carbon electrode; CuO@Cu2O-NW/PVA, cupric/cuprous oxide core shell-nanowire/poly(vinyl alcohol)

### Application in real energy drink samples

**Fig. 7:**
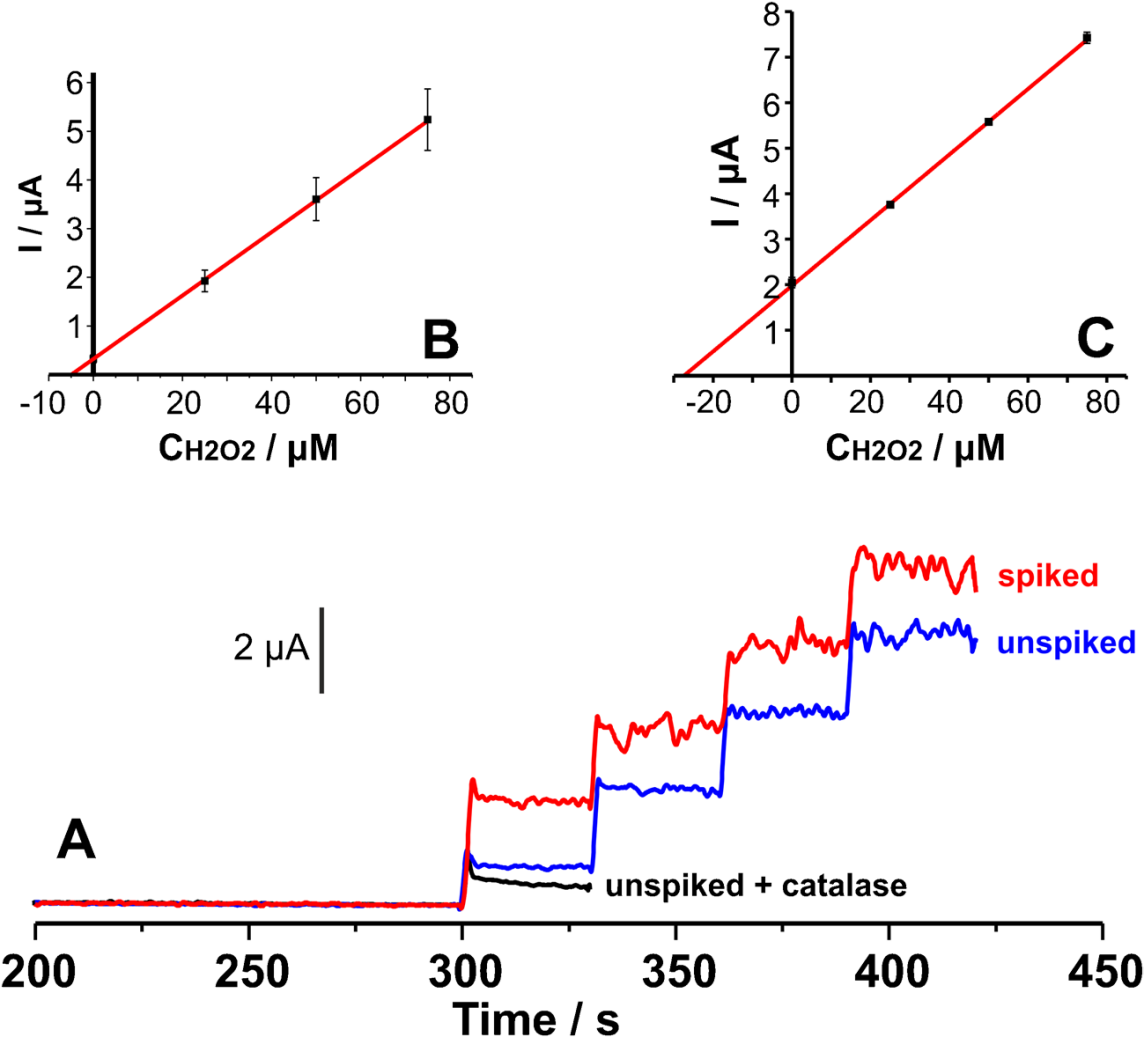
**A** Representative amperometric plots of Co-spark SPEs showing the response in the unspiked sample #1 containing catalase, the unspiked sample #1 plus three additions of 25 μΜ Η_2_Ο_2_, and the sample #1 spiked with 25 μΜ Η_2_Ο_2_ plus three additions of 25 μΜ Η_2_Ο_2_. Standard addition plots for the **B** unspiked and **C** spiked sample #1

The response of Co-spark SPEs to three common compounds present in energy drink like ascorbic acid, glucose, and caffeine, at a concentration of 5 μM, was investigated with amperometric measurements at 0.3 V in 0.5 M NaOH. Even though the response of Co-spark SPEs to glucose and caffeine was nil, ascorbic acid gave a significant amperometric response which hampered the determination of H_2_O_2_. The interference effect of ascorbic acid and the potential interference of other reducing compounds that might exist in real-world samples were effectively addressed by employing dual measurements in the absence and presence of the enzyme catalase. Following the assay protocol described above, the method was applied to the determination of H_2_O_2_ in two commercial energy drinks. The accuracy of the method was evaluated by recovery studies at both samples fortified with 25 μM H_2_O_2_. The concentration of H_2_O_2_ in both the unspiked and spiked samples was determined using the standard addition method (Fig. 7). The responses of Co-spark SPEs before the three additions, were corrected to that obtained in the corresponding unspiked sample in the presence of catalase. Results are shown in Table 3.

**Table 3:** Determination and recovery of Η_2_Ο_2_ in two commercial energy drinks

Sample	H_2_O_2_ added (μM)	H_2_O_2_ determined (μM)	Recovery (%)
1	0	4.69 ± 0.76	-
	25.0	28.0 ± 2.2	94.3 ± 8.2
2	0 25.0	2.30 ± 0.43 24.9 ± 1.9	- 91.2 ± 9.2

Figures show the means and the standard deviation of the data for three different electrodes

## Conclusions

This work employs low-cost and eco-friendly semi-disposable graphite screen-printed electrodes modified with an ease to perform, extremely fast (9 s), liquid-free method based on direct cobalt pin-to-electrode electrical discharge under ambient conditions.

SEM inspection showed that the direct sparking process has a dual effect on the electrode surface generating both low-dimensional micrometre-sized graphite sheets and spherical cobalt-based nanoparticles with an average diameter of 163 ± 73 nm. Interestingly, after the sparking process, the electroactive area of the electrodes increased by 167%, from 0.1126 to 0.1883 cm^2^. Based on the EDS data, the spark-generated nanoparticles represent different oxide cobalt-based species (Co_x_O_y_), which according with the XPS data can be attributed to Co_3_O_4_ spinel type nanostructures. Furthermore, XPS data also indicated the formation of C − Co bonds that probably occurred due to the extremely high temperatures grown locally due to the sparking process.

Cyclic voltammetric studies demonstrated advanced electrocatalytic properties towards the electro oxidation of H_2_O_2_ at alkaline conditions, enabling the amperometric determination of the target over the concentration range 1 − 102 μM (LOD 0.6 μM). Due to the high electrocatalytic properties of spark generated Co_x_O_y_ NPs toward other reducing compounds, potential interferences in real-world samples were mitigated by subtracting the signal obtained from the sample containing catalase. The analytical data obtained from antioxidant-rich real-world samples, such as energy drinks, suggest that the method holds promise for the routine analysis of H_2_O_2_ in various food and drink products with minimal sample preparation.

### Supplementary Information

Below is the link to the electronic supplementary material.

Supplementary file1 (DOCX 255 KB)

## Data Availability

All data generated or analysed during this study are included in this published article and the supplementary information file.
