# Supplementary material for: Eco-friendly spark-generated CoxOy nanoparticle-modified graphite screen-printed sensing surfaces for the determination of H2O2 in energy drinks
Source: Mikrochim Acta. 2024 Feb 22;191(3):150. doi: 10.1007/s00604-024-06233-3 (PMC10884044; doi:10.1007/s00604-024-06233-3)
Supplement: Supplementary file 1 — Supplementary file1 (DOCX 255 KB) [file 604_2024_6233_MOESM1_ESM.docx]

**Supplementary Information**

**Eco-friendly spark-generated Co_x_O_y_ nanoparticle-modified graphite screen-printed sensing surfaces for the determination of H_2_O_2_ in energy drinks**

**Maria Siampani^1^, Alexandros Ch. Lazanas^1*^, Konstantinos Spyrou^2^, Mamas I. Prodromidis^1,*^**

^1^ Department of Chemistry, University of Ioannina, Ioannina 451 10, Greece

^2^ Department of Materials Science & Engineering, University of Ioannina, Ioannina 451 10, Greece

*Corresponding authors. **Tel:** +30−26510−08301

**E−mails**:

[mprodrom@uoi.gr](mailto:mprodrom@uoi.gr) (M.I. Prodromidis)

[alazanas@outlook.com.gr](mailto:alazanas@outlook.com.gr) (A.Ch. Lazanas)

**===========================================**


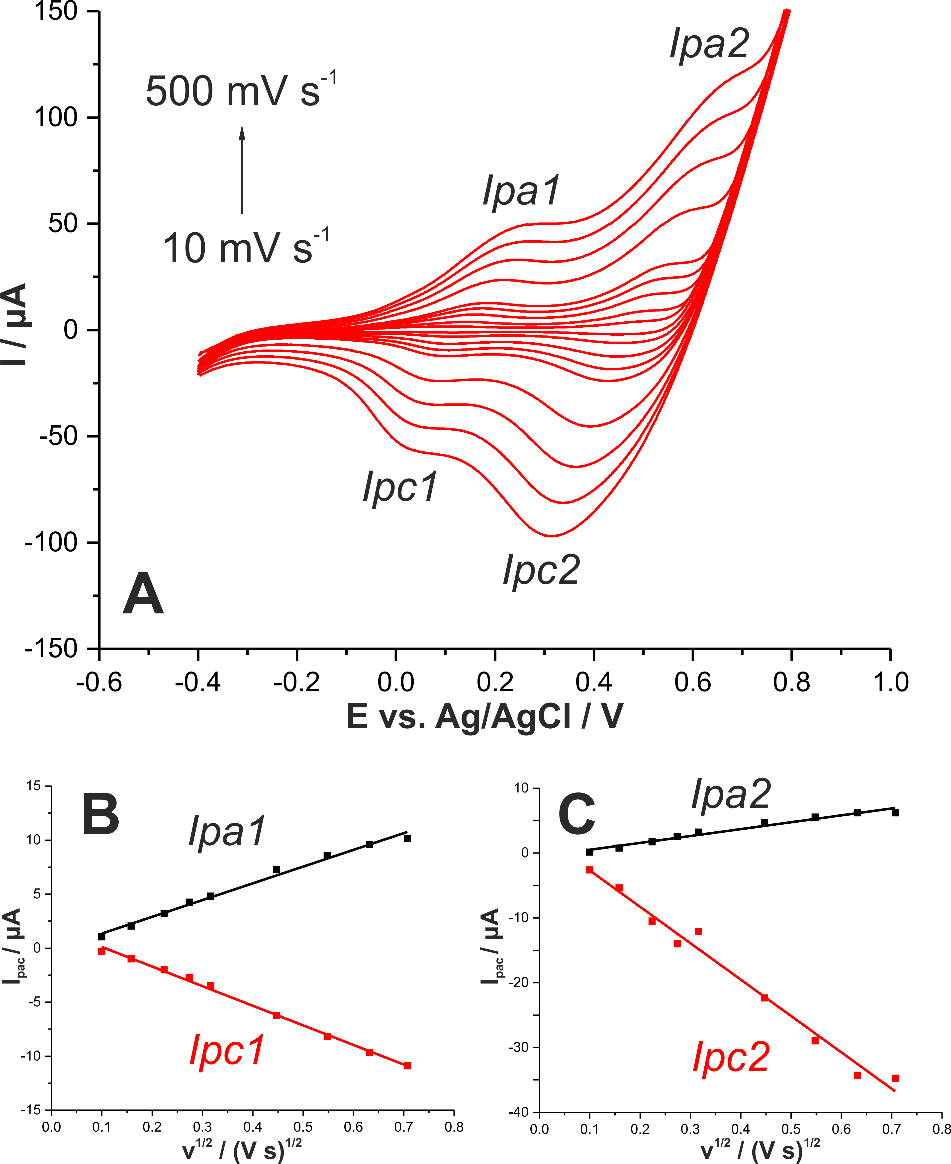


**Fig. S1.** (A) Cyclic voltammograms of Co-spark SPE in 0.5 M NaOH at increasing scan rates: 10, 25, 50, 75, 100, 200, 300, 400, and 500 mV s^−1^. Linear fit of (B) Ip_a_1, Ip_c_1 and (C) Ip_a_2, Ip_c_2 to the square root of scan rate.


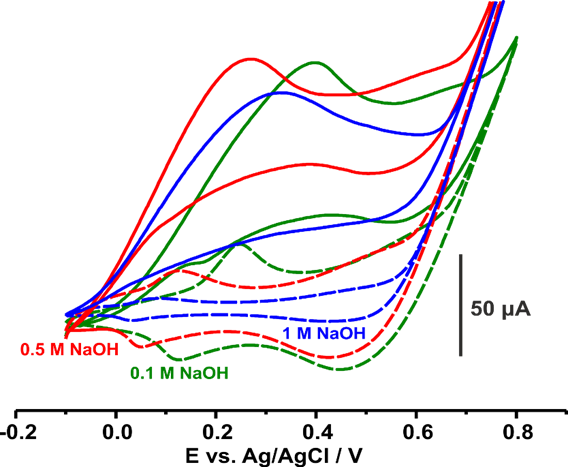


**Fig. S2.** Cyclic voltammograms of Co-spark SPE at (green line) 0.1 M, (red line) 0.5 M and (blue line) 1 M NaOH in the (dashed line) absence and (solid line) presence of 5 mM H_2_O_2_. Scan rate 50 mV s^−1^.
